# CISAT, a CoPP-Induced lncRNA, Improves Cardiac Mesenchymal Progenitor Cell Survival and Myocardial Repair via SFPQ/NRF2/p38 Redox Regulation

**DOI:** 10.3390/cells15060557

**Published:** 2026-03-20

**Authors:** Xiuchun Li, Xiao-Liang Wang, Sofia Lopez, Jill Wang, Chuanxi Cai

**Affiliations:** 1 Department of Surgery, Davis Heart and Lung Research Institute, The Ohio State University Wexner Medical Center, Columbus, OH 43210, USA; 2 Department of Surgery, Division of Surgical Sciences, University of Virginia, Charlottesville, VA 22903, USA

**Keywords:** lncRNA CISAT, cardiac progenitor cells, NRF2 signaling, cellular senescence, myocardial repair

## Abstract

Cellular therapy using human cardiac mesenchymal progenitor cells (hMPCs) for regenerative medicine is hindered by poor cell survival and senescence. Long non-coding RNAs (lncRNAs) are critical regulators of cellular processes, yet their role in cardiac aging remains underexplored. Here, lncRNA microarray profiling identified a novel lncRNA, XLOC_002543, upregulated in hMPCs preconditioned with cobalt protoporphyrin (CoPP), which was named CoPP-Induced and SFPQ-Associated RNA Transcript (CISAT) due to its interaction with splicing factor proline and glutamine rich (SFPQ), confirmed via RNA pull-down and immunoprecipitation. CISAT was the only highly expressed transcript among seven lnc-ANKMY1-5 variants in hMPCs, as shown by RT-PCR. Notably, CISAT expression decreased in aging/senescent hMPCs, correlating with elevated p16^INK4A^, a senescence marker. Overexpression of CISAT reduced p16^INK4A^ levels; enhanced hMPC survival, proliferation, and migration; and increased antioxidant and anti-apoptotic protein expression, while CISAT knockdown reduced resistance to H_2_O_2_-induced oxidative stress. In vivo, intramyocardial transplantation of CISAT-overexpressed hMPCs in an immune-deficient murine myocardial infarction model reduced fibrosis, promoted angiogenesis, and preserved cardiac function. Mechanistically, CISAT interacts with SFPQ to regulate NRF2-mediated redox homeostasis and inhibits p38 MAPK phosphorylation, mitigating senescence and enhancing cell survival. These findings suggest that targeting CISAT to modulate redox signaling and p38 MAPK pathways in aging hMPCs could improve their therapeutic efficacy for myocardial repair in heart disease.

## 1. Introduction

Cardiovascular diseases, including myocardial infarction (MI) and heart failure, remain the leading cause of mortality worldwide [1,2,3], contributing to millions of deaths annually and imposing a significant socioeconomic burden. Despite advances in pharmacological and interventional therapies, the heart’s limited regenerative capacity necessitates innovative approaches to restore myocardial function post-injury. Cellular therapies, particularly those utilizing human cardiac mesenchymal progenitor cells (hMPCs), have emerged as a promising strategy for regenerative medicine due to their potential to differentiate into cardiac lineages and secrete paracrine factors that promote angiogenesis, reduce fibrosis, and enhance tissue repair [4,5]. These properties make hMPCs an attractive candidate for repairing damaged myocardium, particularly in the context of ischemic heart disease. However, the clinical translation of hMPC-based therapies is hindered by several obstacles, including poor donor cell survival in the hostile ischemic microenvironment and accelerated cellular aging or senescence, which impair their regenerative efficacy [6,7].

Cellular senescence, characterized by irreversible cell cycle arrest and the upregulation of markers such as p16^INK4A^, is a major barrier to the therapeutic success of hMPCs [8,9]. In the post-infarct heart, oxidative stress, hypoxia, and inflammation create a challenging environment that exacerbates senescence and apoptosis, reducing the survival, proliferation, and migratory capacity of transplanted cells [8]. Preconditioning strategies, such as treatment with cobalt protoporphyrin (CoPP), a potent inducer of heme oxygenase-1 (HO-1) and the nuclear factor erythroid 2-related factor 2 (NRF2) pathway, have shown promise in enhancing the resilience of cardiac progenitor cells against oxidative stress [10]. CoPP activates antioxidant and anti-apoptotic pathways, improving cell survival and engraftment in preclinical models of myocardial infarction [10]. However, the molecular mechanisms underlying these protective effects, particularly in the context of hMPC senescence, remain incompletely understood, necessitating further exploration of novel regulatory pathways.

Long non-coding RNAs (lncRNAs), a class of non-coding RNAs longer than 200 nucleotides [11,12], have recently gained attention for their critical roles in regulating diverse biological processes, including cell survival, proliferation, and senescence, across various cell types [13]. In the cardiovascular system, lncRNAs such as MALAT1 and H19 have been implicated in modulating oxidative stress responses, angiogenesis, and cardiac repair, often through interactions with RNA-binding proteins or epigenetic complexes [14,15]. Despite these advances, the role of lncRNAs in cardiac aging and senescence, particularly in hMPCs, remains in its infancy. Emerging evidence suggests that lncRNAs may serve as key regulators of redox homeostasis and signaling pathways like p38 MAPK, which are critical for mitigating senescence and enhancing the therapeutic potential of progenitor cells [16,17]. Identifying specific lncRNAs that modulate these processes in hMPCs could unlock new strategies to overcome the limitations of cellular therapies for heart disease.

In this study, we report the discovery of a novel lncRNA, termed CoPP-Induced and SFPQ-associated RNA transcript (CISAT), identified through lncRNA microarray profiling in hMPCs preconditioned with CoPP. CISAT, a transcript of the lnc-ANKMY1-5 gene, is significantly upregulated by CoPP and interacts with the splicing factor proline and glutamine rich (SFPQ) to modulate NRF2-related redox homeostasis and inhibit p38 MAPK signaling, thereby reducing senescence and promoting cell survival. Our findings demonstrate that CISAT expression is decreased in aging hMPCs, correlating with elevated p16^INK4A^, and that its overexpression enhances hMPC functionality and myocardial repair in a murine model of myocardial infarction. By elucidating the role of CISAT in regulating redox and senescence pathways, this study aims to provide a foundation for targeted lncRNA-based interventions to improve the efficacy of hMPC therapies for patients with heart disease.

## 2. Materials and Methods

### 2.1. Reagents

Ham’s F12 medium was purchased from Invitrogen (Carlsbad, CA, USA). Fetal bovine serum (FBS) was procured from Hyclone (Logan, UT, USA). Primers for quantitative PCR targeting specific genes were obtained from Integrated DNA Technologies (Coralville, IA, USA) and are detailed in Supplementary Table S1. Primary antibodies used are listed in Supplementary Table S2. Unless otherwise specified, all chemicals were purchased from Sigma-Aldrich (St. Louis, MO, USA).

### 2.2. hMPC Cell Culture

Human cardiac mesenchymal progenitor cells (hMPCs) were previously established in our laboratory using flow cytometry-based cell sorting, selecting for cells with low mitochondrial membrane potential, as described [18]. The isolation was conducted under Institutional Review Board protocol (IRB#3728) approved by Albany Medical College, with written informed consent obtained from patients. hMPCs were maintained in growth medium comprising Ham’s F12 medium (Invitrogen), 10% FBS (Hyclone), 10 ng/mL human basic fibroblast growth factor (bFGF, Sigma-Aldrich), 0.005 U/mL human erythropoietin (EPO, Sigma-Aldrich), 0.2 mM L-glutathione (Sigma-Aldrich), and 100 U/mL penicillin with 100 µg/mL streptomycin (Gibco, Thermo Fisher Scientific, Waltham, MA, USA). Cultures were maintained at 37 °C in a humidified atmosphere with 5% CO_2_ and 5% O_2_.

### 2.3. Lentiviral Production and hMPC Transduction

Bacterial stocks containing the open reading frame (ORF) expression clone for CISAT (Catalog #: VB170424-1144-Stbl3 from Cyagen Biosciences, Santa Clara, CA, USA) and four short hairpin RNA (shRNA) clones targeting human XLOC_002543/CISAT (Catalog# CS-SH182T-LVRU6GP-01 from GeneCopoeia, Inc., Rockville, MD, USA) were used to generate plasmid DNA with the Qiagen Maxiprep Kit (Qiagen, Hilden, Germany). The ORF expression clone for SFPQ (NM_005066.2) was generated by inserting its cDNA into the lentiviral backbone of the Lv206 plasmid (Catalog #EX-A6434-Lv206), which also expresses mCherry to enable monitoring of transduction efficiency. For lentiviral production, HEK293FT cells were seeded in T-75 flasks 24 h prior to transfection. At 70% confluence, cells were co-transfected with the target plasmid, psPAX2, and pMD2.G packaging plasmids at a 4:3:1 ratio using GeneJammer transfection reagent (Agilent Technologies, Santa Clara, CA, USA). After 18–24 h, the medium was replaced with fresh growth medium. Supernatants containing lentiviral particles were collected at 48 and 72 h post-transfection, and viral presence was confirmed using Lenti-X GoStix (Takara Bio, Kusatsu, Japan). Lentiviral stocks were concentrated 25-fold using the Lenti-X Concentrator (Takara Bio).

For transduction, hMPCs were seeded in 6-well plates and cultured for 24 h to reach 70% confluence. The medium was supplemented with 5 µg/mL Polybrene and lentiviral particles, followed by gentle mixing. After 18–24 h, the medium was replaced with fresh growth medium. Control groups were transduced with empty vector lentiviral particles.

### 2.4. p38 MAPK Inhibition

Following lentiviral transduction as described above, hMPCs were treated with 10 µM SB203580 (p38-specific inhibitor) or vehicle control 18–24 h post-transduction. The inhibitor was applied for 24 h, and p38 inhibition was verified by Western blot analysis, as previously described [10].

### 2.5. Senescence-Associated Beta-Galactosidase Assay

Cell senescence was assessed using the Senescence-Associated β-Galactosidase (SA-β-Gal) Staining Kit (Cell Signaling Technology, Danvers, MA, USA). After 48 h of lentiviral-mediated CISAT overexpression, hMPCs were washed twice with phosphate-buffered saline (PBS), fixed, and stained according to the manufacturer’s protocol. Cells were incubated with β-galactosidase staining solution at 37 °C for 24 h. SA-β-Gal-positive cells, identified by blue staining, were visualized under a microscope at 200× magnification.

### 2.6. Flow Cytometry-Based Apoptosis Assay

Apoptosis and necrosis were quantified using the Annexin V/Propidium Iodide (PI) Apoptosis Detection Kit (Invitrogen). At 36–48 h post-lentiviral transduction or 24 h post-SB203580 treatment, hMPCs were trypsinized and resuspended in serum-free Ham’s F12 medium containing 1 mM H_2_O_2_ for 90 min. Cells were then washed in PBS, resuspended in 50 µL Annexin-binding buffer with 1 µg/mL PI and 1 µL Alexa Fluor 647-Annexin V (Invitrogen), and incubated for 15 min at room temperature. After adding 100 µL Annexin-binding buffer, samples were analyzed using the Guava EasyCyte System (EMD Millipore, Burlington, MA, USA) [10]. Data was processed with GuavaSoft Module software (version 3.1.1). Apoptotic cells were identified as Annexin V-positive/PI-negative (Q4) or Annexin V/PI-double-positive (Q2), necrotic cells as PI-positive only (Q1), and viable cells as double-negative (Q3).

### 2.7. Cell Proliferation Assay (BrdU Incorporation)

Cell proliferation was evaluated by BrdU incorporation, as previously described [10]. Following 18–24 h of lentiviral transduction for CISAT overexpression or knockdown, hMPCs were incubated in growth medium supplemented with 10 µM BrdU for 36–48 h. Cells were then trypsinized, fixed in 4% paraformaldehyde, treated with 2 M HCl for 30 min, and permeabilized with 0.5% Triton X-100 for 20 min. After blocking in 2% BSA in PBS for 30 min, cells were stained with anti-BrdU antibody (Life Technologies, Carlsbad, CA, USA) for 2 h, followed by Alexa Fluor 647-conjugated secondary antibody (Life Technologies) for 1 h at room temperature. Samples were analyzed by flow cytometry using the Guava EasyCyte System.

### 2.8. Measurement of Reactive Oxygen Species (ROS)

ROS levels were measured using the Dihydroethidium (DHE) Cellular ROS Detection Assay Kit (Invitrogen). At 36–48 h post-lentiviral transduction, hMPCs were trypsinized, washed with PBS, and stained with 10 µM DHE for 30 min at 37 °C in the dark. Fluorescence intensity was quantified using the Guava EasyCyte System.

### 2.9. RNA Isolation and Quantitative PCR

Total RNA was extracted using the Aurum Total RNA Mini Kit (Bio-Rad, Hercules, CA, USA) and quantified with a Nanodrop 2000C spectrophotometer (Thermo Fisher Scientific). cDNA was synthesized using the iScript cDNA Synthesis Kit (Bio-Rad). Quantitative PCR was performed with iQ SYBR Green Supermix (Bio-Rad) and primers from Integrated DNA Technologies, using a Bio-Rad iQ5 optical module. PCR conditions included initial denaturation at 95 °C for 2 min, followed by 40 cycles of 95 °C for 15 s and 60 °C for 40 s. GAPDH served as the internal control. Primer sequences are listed in Supplementary Table S1.

### 2.10. Western Blot Analysis

Western blotting was performed as previously described [10]. Cells were washed twice with ice-cold PBS and lysed in modified immunoprecipitation assay buffer (150 mM NaCl, 5 mM EDTA, 1% Nonidet P-40, 20 mM Tris-HCl, pH 7.5) supplemented with protease and phosphatase inhibitors (Sigma-Aldrich). Proteins (15–20 µg) were separated on 12% SDS-PAGE gels and transferred to nitrocellulose membranes. After blocking with 5% non-fat milk in TBST (50 mM Tris-HCl, pH 7.4, 150 mM NaCl, 0.1% Tween 20) for 1 h, membranes were incubated with primary antibodies overnight, followed by HRP-conjugated secondary antibodies (1:4000; Cell Signaling Technology) for 1.5 h. Chemiluminescence was detected using SuperSignal West Femto Substrate (Thermo Fisher Scientific) and imaged with the ImageQuant LAS 3000 system (GE Healthcare, Chicago, IL, USA). GAPDH served as the loading control, and band densities were quantified using ImageJ software (version 1.54). Antibodies are listed in Supplementary Table S2.

### 2.11. Mouse Model of Acute Myocardial Infarction and Cell Delivery

All in vivo experiments adhered to National Institutes of Health Guidelines and were approved by The Ohio State University Institutional Animal Care and Use Committee. NOD.Cg-Prkdcscid/Il2rgtm1Wjl/SzJ (NSG) mice (10–12 weeks old) were obtained from The Jackson Laboratory (Bar Harbor, ME, USA). Acute myocardial infarction was induced by ligating the left anterior descending coronary artery for 60 min, followed by reperfusion. At 45 min post-reperfusion, hMPCs (5 × 10^5^ cells in 40 µL), RAACs, or vehicle were injected intramyocardially using a 30-gauge needle, as described [18]. Mice were monitored for 39 days post-surgery, with survival rates of ~90% at 24 h and >85% at 35 days.

### 2.12. Echocardiographic Analysis

M-mode echocardiography was performed as previously described [18] using the Vevo 3100 Imaging Platform (VisualSonics, Toronto, ON, Canada) under 1–2% isoflurane anesthesia. Images were acquired at baseline (7 days before surgery) and at 5 and 35 days post-hMPC administration. Data were analyzed offline by investigators blinded to treatment groups.

### 2.13. Heart Section Staining

Paraffin-embedded 5-µm heart sections were deparaffinized in xylene, rehydrated through graded ethanol (100%, 95%, 70%), and subjected to antigen retrieval. Immunohistochemical staining was performed as previously described [18], and images were captured using a Leica DMI 4000B confocal microscope (Leica Microsystems, Deerfield, IL, USA). Quantitative analysis was conducted with ImageJ software (NIH). At least six hearts per group were analyzed, with three left ventricular (LV) sections per heart (100–120 µm intervals below the ligation site).

Apoptosis was assessed using the TUNEL assay (GeneCopoeia, Rockville, MD, USA), and cardiac fibrosis was evaluated with wheat germ agglutinin (WGA) staining (Invitrogen), following manufacturer protocols.

### 2.14. Statistical Analysis

Data are expressed as means ± SEM from at least three independent experiments or over 6 animals per group. Statistical significance was determined using Student’s *t*-test for two-group comparisons or one-way ANOVA for multiple groups, with a *p*-value < 0.05 considered significant.

## 3. Results

### 3.1. Identification of lncRNA CISAT as a Key Responder to CoPP Preconditioning in hMPCs

**Figure 1:**
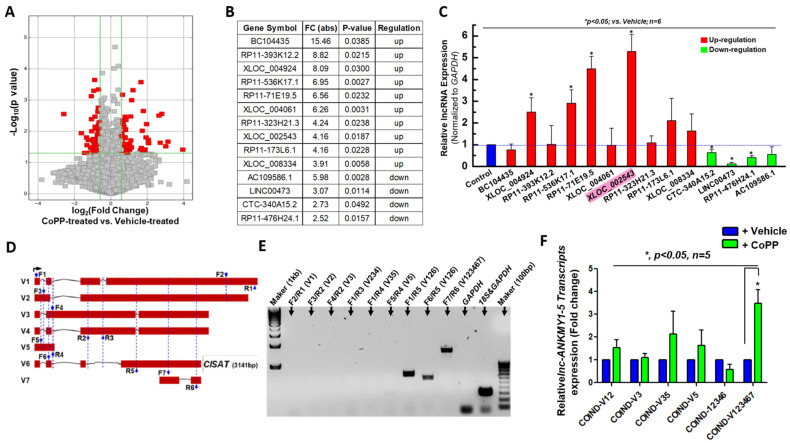
Identification and validation of XLOC_002543 as a highly upregulated lncRNA in hMPCs following CoPP Preconditioning. (**A**) Volcano plot illustrating differentially expressed long non-coding RNAs (lncRNAs) (red dots) in human cardiac mesenchymal progenitor cells (hMPCs) after cobalt protoporphyrin (CoPP) treatment, with XLOC_002543 identified as the top upregulated lncRNA. (**B**) Table listing the top 10 upregulated and top 4 downregulated lncRNAs based on microarray analysis of hMPCs treated with CoPP. (**C**) Real-time quantitative PCR (qPCR) validation of differentially expressed lncRNAs listed in panel B, with XLOC_002543 highlighted with light red as the most significantly upregulated lncRNA after normalized to their expression in vehicle treated sample (*blue column*). (**D**) Schematic representation of seven transcript variants (V1–V7) of the lncRNA ANKMY1-5 gene, with the locations of forward (F) and reverse (R) primers for qPCR indicated by blue arrows. (**E**) qPCR results using various primer combinations, confirming that transcript variant #6 (V6, CISAT) is highly expressed in hCPCs, as determined by primer specificity and PCR product size. (**F**) Quantitative real-time PCR analysis of relative mRNA expression levels of different XLOC_002543 transcript variants in hMPCs with CoPP treatment, showing significant upregulation of transcript #6 (V6, CISAT). The y-axis represents mRNA expression levels normalized to GAPDH. Data are presented as mean ± SEM. * *p* < 0.05, unpaired Student’s *t*-test, *n* ≥ 3.

Cobalt protoporphyrin (CoPP), a well-recognized inducer of heme oxygenase 1, facilitates endogenous carbon monoxide production and confers protection against ischemia/reperfusion injury in multiple vital organs [19,20,21]. Our prior research demonstrated that CoPP mitigates apoptosis in human cardiac progenitor cells (hCPCs) and enhances their therapeutic efficacy post-transplantation in a murine model of myocardial infarction [22]. To investigate downstream long non-coding RNAs (lncRNAs) potentially associated with CoPP treatment in human cardiac mesenchymal progenitor cells (hMPCs) that were previously established in our lab [18], we conducted lncRNA microarray screening, which identified a range of differentially expressed genes in CoPP-treated hMPCs (Figure 1A,B). Quantitative real-time PCR analysis validated these findings and revealed lncRNA CISAT (XLOC_002543) as the most significantly upregulated gene associated with CoPP preconditioning (Figure 1C). Genomic analysis indicated that XLOC_002543 comprises seven versions of distinct transcript variants. To determine the predominant transcript in hMPCs, we designed specific primer pairs (Figure 1D). PCR results demonstrated that only primer pairs F1/R5, F6/R5, and F7/R6 yielded amplification products, indicating that transcript variant version six is the most abundantly expressed in hMPCs (Figure 1E). Furthermore, expression analysis of XLOC_002543 transcripts in CoPP-treated hMPCs confirmed that only the sixth version of XLOC_002543 transcripts exhibited significant upregulation (Figure 1F). Thus, the sixth version of XLOC_002543 transcripts, designated as CISAT, represents the primary lncRNA responsive to CoPP treatment in hMPCs.

### 3.2. Enhanced Cell Survival, Proliferation, Migration, and Reduced ROS Generation in hMPCs with Overexpressed lncRNA CISAT

**Figure 2:**
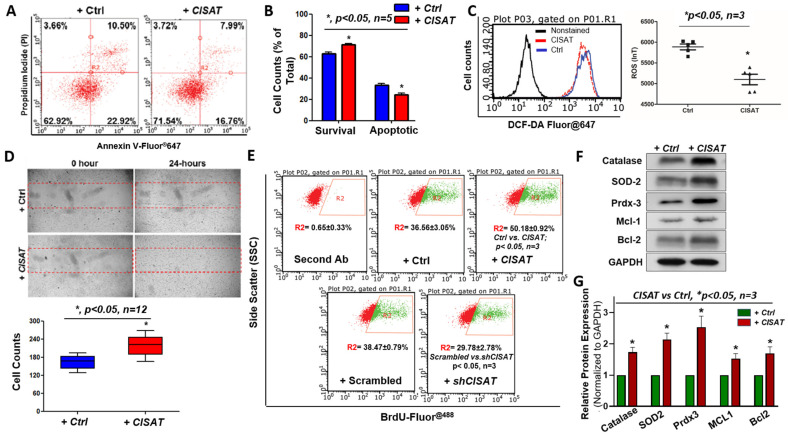
Overexpression of CISAT Enhances Cell Survival, Proliferation, and Migration while Reducing Reactive Oxygen Species (ROS) Generation in hCPCs. (**A**) Flow cytometry analysis of apoptosis in CISAT-overexpressed human cardiac progenitor cells (hCPCs) using Annexin V-Fluor^®^ 647 and propidium iodide (PI) double staining. The y-axis represents PI fluorescence, and the x-axis represents Annexin V-Fluor^®^ 647 fluorescence. (**B**) Quantitative analysis of apoptosis data from panel A. (**C**) Flow cytometry analysis of ROS generation in CISAT-overexpressed hCPCs using DCFDA-Fluor^®^ 647 dye staining. (**D**) Representative images from a wound healing assay demonstrating enhanced cell migration in CISAT-overexpressed hCPCs compared to vector control. (**E**) Bromodeoxyuridine (BrdU) fluorescence staining followed by flow cytometry analysis to assess cell proliferation in hCPCs with CISAT overexpression or knockdown. (**F**) Western blot analysis showing that CISAT overexpression promotes hCPC survival, associated with upregulation of anti-apoptotic and anti-oxidative gene expression. (**G**) Quantitative analysis of Western blot data from panel F. Data are presented as mean ± SEM. * *p* < 0.05, unpaired Student’s *t*-test, *n* ≥ 3.

The therapeutic application of adult cardiac progenitor cells is hindered by challenges such as poor survival, low retention, and limited engraftment in vivo [8]. To elucidate the role of lncRNA CISAT in hMPCs, we conducted a series of functional assays with the gain or loss of CISAT, which overexpression or knocking down were confirmed by qPCR (Supplementary Figure S1). An apoptosis assay using Annexin V and propidium iodide (PI) staining, followed by flow cytometry analysis, was performed to assess cell survival under oxidative stress induced by 1.5-h treatment with 2 mM H_2_O_2_. Results showed that hMPCs overexpressing CISAT exhibited a significantly higher proportion of viable cells (71.54 ± 1.42%) compared to the control group (62.92 ± 2.39%), with a corresponding 9% reduction in apoptotic cells (Figure 2A,B). Conversely, the knockdown of CISAT in hMPCs reduced cell viability under H_2_O_2_ stress, consistent with the overexpression findings (Supplementary Figure S2). Given the critical role of reactive oxygen species (ROS) in cellular signaling and cycling, we investigated CISAT’s impact on ROS generation using DCF-DA fluorescence staining and flow cytometry. Overexpression of CISAT significantly reduced ROS levels in hMPCs (Figure 2C). Additionally, cell migration, a key factor in the efficacy of cardiac progenitor cell therapy for ischemic heart diseases, was evaluated using a wound-healing scratch assay, which demonstrated enhanced migration in CISAT-overexpressing hMPCs (Figure 2D). Cell proliferation was assessed via BrdU staining and flow cytometry, revealing that CISAT-overexpressing hMPCs exhibited significantly higher proliferation rates (50.18 ± 0.92%) compared to controls (36.56 ± 3.05%) (Figure 2E). Knockdown of CISAT, in contrast, significantly reduced hMPC proliferation (Figure 2E). To explore the mechanisms underlying CISAT’s effects on cell survival and ROS reduction, Western blotting was performed to quantify anti-apoptotic and antioxidant proteins. Overexpression of CISAT significantly increased the levels of Bcl-2, Mcl-1, Catalase, Peroxiredoxin 3 (Prdx3), and SOD2 in hMPCs (Figure 2F,G). Collectively, these findings indicate that CISAT overexpression enhances hMPC survival, proliferation, and migration while reducing ROS production, highlighting its potential therapeutic significance.

### 3.3. Association of lncRNA CISAT with Senescence in hMPCs

**Figure 3:**
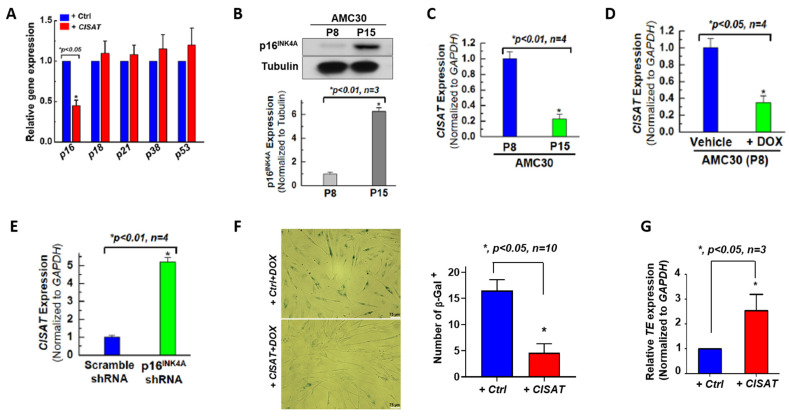
Role of CISAT in hCPC Senescence. (**A**) Quantitative real-time PCR (qPCR) analysis of senescence-associated genes, demonstrating significant downregulation of p16^INK4A^ in human cardiac progenitor cells (hCPCs) overexpressing CISAT. (**B**) Western blot analysis of p16^INK4A^ protein expression in hCPCs subjected to replicative senescence, confirming reduced levels in CISAT-overexpressing cells. (**C**) qPCR analysis revealing a significant decrease in lncRNA CISAT expression in hCPCs following replicative senescence. (**D**) Downregulation of CISAT expression in hCPCs under oxidative stress-induced senescence triggered by doxorubicin treatment, as assessed by qPCR. (**E**). qPCR analysis showing a significant increase in CISAT mRNA expression in hCPCs following knockdown of p16^INK4A^. (**F**) Representative images of senescence-associated β-galactosidase staining in hCPCs overexpressing CISAT and treated with doxorubicin, indicating altered senescence phenotypes. (**G**) qPCR analysis of telomerase (TE) mRNA expression, demonstrating a significant increase in TE levels in CISAT-overexpressing hCPCs. Data are presented as mean ± SEM. * *p* < 0.05, unpaired Student’s *t*-test, *n* ≥ 3.

Aging is an inevitable biological process and a primary risk factor for cardiovascular diseases. Compared to younger human cardiac mesenchymal progenitor cells (hMPCs), aged hMPCs exhibit reduced proliferation and survival [9]. To investigate the role of lncRNA CISAT in the senescence of hMPCs, we evaluated the expression of senescence-associated genes, including p16, p18, p21, p38, and p53, in hMPCs overexpressing CISAT. Quantitative real-time PCR analysis revealed a significant reduction in p16 expression in CISAT-overexpressing hMPCs (Figure 3A). Western blotting confirmed that serial passaging of hMPCs induced senescence, as evidenced by elevated p16 protein levels, corroborated by real-time PCR (Figure 3B,C). These findings suggest that CISAT expression is inversely associated with hMPC senescence, with lower CISAT levels observed in aged hMPCs. To further explore this relationship, hMPCs were treated with doxorubicin to induce senescence or subjected to p16 knockdown (Supplementary Figure S3). As anticipated, doxorubicin treatment reduced CISAT expression, while p16 knockdown, which rejuvenates hMPCs, led to upregulated CISAT expression (Figure 3D,E). To assess whether CISAT could mitigate senescence, we performed senescence-associated β-galactosidase staining on control and CISAT-overexpressing hMPCs following 18-h treatment with 5 μM doxorubicin. Results showed reduced β-galactosidase staining in CISAT-overexpressing hMPCs, indicating a reversal of senescence and a shift toward a younger cellular phenotype (Figure 3F). These data suggest that CISAT overexpression may rejuvenate aged hMPCs, offering a potential strategy to enhance the efficacy of cellular therapies for aging cardiac progenitor cells. Additionally, telomeres, repetitive nucleotide sequences at chromosome ends, serve as a key indicator of cellular aging. Quantitative real-time PCR analysis demonstrated that CISAT significantly increased telomere-related gene expression (Figure 3G), providing further evidence of its role in counteracting hMPC senescence.

### 3.4. Physical Interaction of lncRNA CISAT with SFPQ in the Nucleus of hMPCs

**Figure 4:**
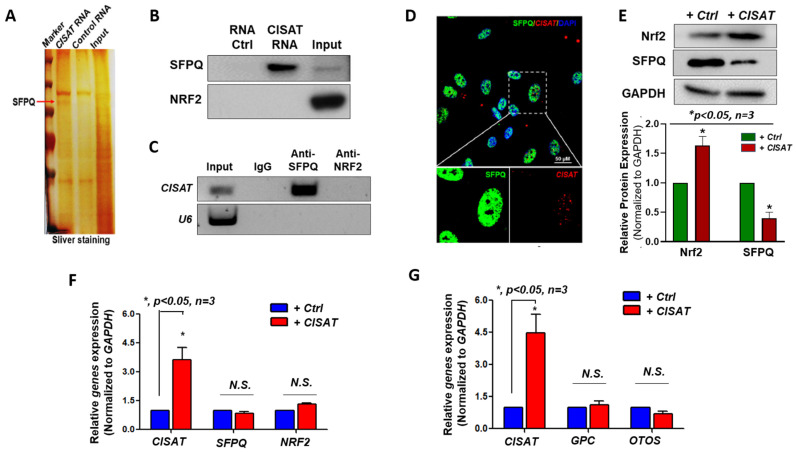
Physical interaction of CISAT with SFPQ in the nucleus of hMPCs. (**A**) Silver staining of proteins pulled down by lncRNA CISAT, with SFPQ identified as one of the CISAT-associated proteins through RNA pulldown and mass spectrometry analysis. (**B**) Western blot analysis confirms that SFPQ, but not NRF2, is specifically pulled down by CISAT lncRNA. (**C**) RNA immunoprecipitation (RIP) assay demonstrates that CISAT is immuno-precipitated by an anti-SFPQ antibody, but not by an anti-NRF2 antibody. (**D**) RNA fluorescence in situ hybridization (FISH) showing co-localization of CISAT and SFPQ within the nucleus of hMPCs. (**E**) Western blot analysis indicates that CISAT overexpression significantly increases NRF2 protein levels and decreases SFPQ protein levels in hMPCs. (**F**) Quantitative real-time PCR (qPCR) analysis of relative mRNA expression of SFPQ and NRF2 in hMPCs following CISAT overexpression, with expression levels normalized to GAPDH. (**G**) qPCR analysis of relative mRNA expression of CISAT neighboring genes, including GPC and OTOS, in hMPCs overexpressing CISAT, with expression levels normalized to GAPDH. Data are presented as mean ± SEM. * *p* < 0.05, unpaired Student’s *t*-test, *n* ≥ 3.

To understand how CISAT works in hMPCs, RNA in situ hybridization (RNA-ISH) was conducted, and the results revealed that CISAT is predominantly localized in the nucleus of hMPCs (Supplementary Figure S4). To elucidate the molecular mechanisms underlying CISAT’s functions, we employed an RNA–protein interaction strategy to identify proteins that interact with CISAT. A CISAT pulldown assay, followed by silver staining, identified a specific band at approximately 100 kDa (Figure 4A). Proteomic mass spectrometry analysis of this band highlighted candidate proteins, including SFPQ (Splicing Factor Proline and Glutamine Rich) and NRF2, for further validation. Subsequent CISAT pulldown and RNA immunoprecipitation (RIP) assays confirmed that CISAT specifically interacts with SFPQ, but not NRF2 (Figure 4B,C). Immunofluorescence staining further supported this interaction by demonstrating co-localization of CISAT and SFPQ in the nucleus of hMPCs (Figure 4D). Intriguingly, CISAT overexpression significantly reduced SFPQ protein levels while increasing NRF2 protein levels, without altering their respective mRNA levels, as assessed by quantitative real-time PCR (Figure 4E,F). These results suggest that SFPQ and NRF2 may play critical roles in mediating CISAT’s functions in hMPCs. Additionally, we investigated whether CISAT regulates neighboring genes, such as GPC and OTOS, and found no significant changes in their expression via quantitative real-time PCR (Figure 4G). Collectively, these findings establish that CISAT physically interacts with SFPQ, downregulates SFPQ protein levels, and upregulates NRF2 protein expression, a key transcription factor regulating cellular redox homeostasis, highlighting their potential significance in CISAT-mediated effects in hMPCs.

### 3.5. Cytoprotective Role of lncRNA CISAT in hMPCs via the SFPQ/p-p38/NRF2 Signaling Axis

**Figure 5:**
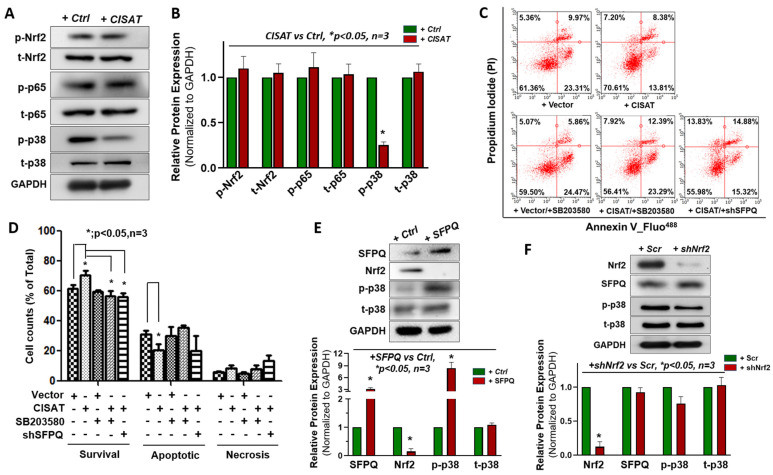
Cytoprotective role of CISAT in hMPCs via the SFPQ/p-p38/NRF2 signaling pathway. (**A**) Representative Western blot images showing the expression levels of total and phosphorylated p38 (p-p38), phosphorylated NRF2 (p-NRF2), and phosphorylated p65 (p-p65) in hMPCs overexpressing CISAT. (**B**) Quantitative analysis of protein expression levels from panel A. (**C**) Annexin V/PI staining followed by flow cytometry (FACS) analysis of hMPCs with overexpressed vector or CISAT, with or without SFPQ knockdown or treatment with SB203580, subjected to oxidative stress induced by 2 mM H_2_O_2_ for 1.5 h. (**D**) Quantitative analysis of apoptosis data from panel C. (**E**) Western blot analysis of total and phosphorylated p38, NRF2, and SFPQ in hMPCs overexpressing SFPQ. (**F**) Western blot analysis of phosphorylated p38, NRF2, and SFPQ in hMPCs with NRF2 knockdown. Data are presented as mean ± SEM. * *p* < 0.05 versus vector Ctrl, unpaired Student’s *t*-test, *n* ≥ 3.

To further elucidate the molecular mechanisms driving the cytoprotective effects of lncRNA CISAT in human cardiac mesenchymal progenitor cells (hMPCs), we investigated several signaling pathways potentially critical for CISAT-mediated cell survival, including NF-κB, NRF2, and p38 MAPK pathways. Our findings revealed that CISAT overexpression significantly reduced p38 phosphorylation, indicating suppression of the p38 MAPK signaling pathway as a central mechanism for CISAT’s cytoprotective effects in hMPCs (Figure 5A,B). Other pathways, including NF-κB pathway, showed no significant involvement (Figure 5A,B). To validate the role of p38 MAPK signaling, we treated hMPCs with the p38-specific inhibitor SB203580, which markedly diminished the cytoprotective effect of CISAT overexpression, as evidenced by reduced cell viability (control: 61.36 ± 2.75%; CISAT overexpression: 70.61 ± 3.58%; CISAT overexpression with SB203580: 56.41 ± 3.56%; Figure 5C,D). These results confirm the pivotal role of p38 MAPK signaling in CISAT’s cytoprotective function. Additionally, simultaneous CISAT overexpression and SFPQ knockdown abolished CISAT’s cytoprotective effects, underscoring the critical regulatory role of the CISAT-SFPQ interaction in enhancing hMPC survival (Figure 5C,D). Consistent with these findings, CISAT overexpression decreased SFPQ protein levels, increased NRF2 protein levels, and inhibited p38 MAPK signaling (Figure 4E and Figure 5A). To dissect the interplay among SFPQ, NRF2, and p38, we overexpressed SFPQ in hMPCs using lentiviral transduction. Western blotting revealed that SFPQ overexpression activated p38 MAPK signaling and significantly reduced NRF2 protein levels (Figure 5E), whereas NRF2 knockdown had no effect on SFPQ levels (Figure 5F). These data highlight the importance of SFPQ balance in CISAT’s function: SFPQ knockdown impairs CISAT’s cytoprotective effects, while SFPQ overexpression activates p38 MAPK signaling, counteracting CISAT’s inhibition of this pathway. Mechanistically, CISAT interacts with SFPQ, downregulating its protein levels, which suppresses p38 MAPK signaling, enhances NRF2 transcription, and upregulates anti-apoptotic (BCL2, MCL1) and antioxidant proteins (Catalase, SOD2, PRDX3). This molecular cascade protects hMPCs from apoptosis and reduces ROS generation, establishing the SFPQ/p-p38/NRF2 axis as a key mediator of CISAT’s cytoprotective effects.

### 3.6. Transplantation of CISAT-Overexpressing hMPCs Preserves Cardiac Function in a Mouse Model of MI

**Figure 6:**
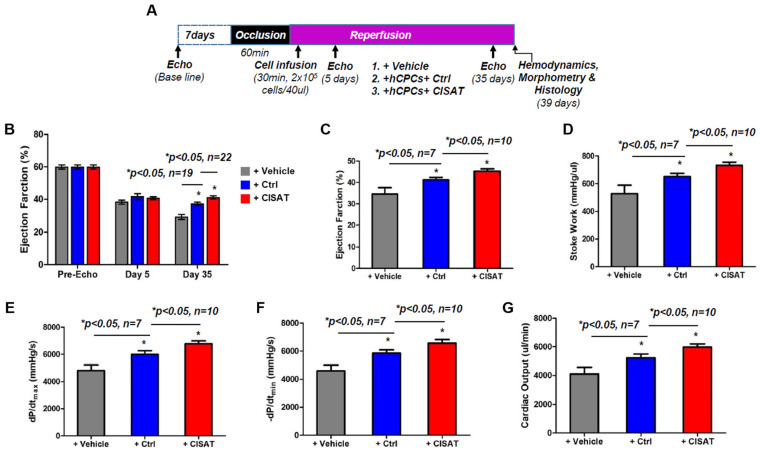
Implantation of CISAT-Overexpressing hMPCs preserves cardiac function in a murine model of MI. (**A**) Schematic representation of the experimental protocol for inducing MI in a murine model and administering cellular therapy. (**B**) Echocardiographic analysis of ejection fraction (EF) in MI mice treated with vehicle (n = 7), control hMPCs (Ctrl, n = 8), or CISAT-overexpressing hMPCs (n = 10). (**C**–**G**) Hemodynamic parameter analyses, including ejection fraction (EF) (**C**), stroke volume (**D**), maximum rate of pressure change (dP/dt max) (**E**), minimum rate of pressure change (negative dP/dt min) (**F**), and cardiac output (**G**) in MI mice treated with vehicle (n = 7), control hMPCs (n = 8), or CISAT-overexpressing hMPCs (n = 10) at 35 days post-MI. Data are presented as mean ± SEM. * *p* < 0.05, as calculated using one-way ANOVA followed by Tukey’s multiple comparison test.

To evaluate whether CISAT overexpression enhances the therapeutic potential of hMPCs in vivo, we transduced hMPCs with CISAT lentivirus and transplanted these cells into a severe combined immunodeficient (SCID) mouse model of ischemia/reperfusion (I/R)-induced myocardial infarction (MI). Cardiac function was assessed over 35 days to determine the efficacy of cellular therapy for cardiac repair. Briefly, baseline echocardiographic recordings of heart function were obtained 7 days prior to surgery. The left anterior descending (LAD) artery was ligated for 60 min, and 2 × 10^5^ hMPCs (control or CISAT-overexpressing) or vehicle were then injected into the risk area after 30 min of reperfusion, after which echocardiographic assessments were conducted at 5 and 35 days post-transplantation, and hearts were collected on day 39 for analysis (Figure 6A). M-mode echocardiograms revealed significantly preserved cardiac function in both the hMPCs+Ctrl and hMPCs+CISAT groups compared to the vehicle group, with the hMPCs+CISAT group outperforming the hMPCs+Ctrl group (Figure 6B). The study included 19 mice in the hMPCs+Ctrl group (9 males, 10 females) and 23 mice in the hMPCs+CISAT group (13 males, 10 females). At 5 days post-I/R, left ventricular (LV) function, measured by left ventricular ejection fraction (LVEF), was comparable across groups (vehicle: 38.20 ± 1.25%; hMPCs+Ctrl: 41.62 ± 1.81%; hMPCs+CISAT: 40.48 ± 0.91%) and reduced compared to baseline (59.82 ± 1.22%), confirming LV dysfunction post-I/R (Figure 6B). By day 35, LV dysfunction worsened in the vehicle group, whereas the hMPCs+Ctrl group exhibited significantly better LVEF (37.20 ± 0.96%) compared to the vehicle group (29.43 ± 1.51%). Notably, the hMPCs+CISAT group showed a further significant higher level of LVEF (42.12 ± 0.97%) compared to the hMPCs+Ctrl group (Figure 6B). These results indicate that both hMPCs and CISAT-overexpressing hMPCs prevent further deterioration of cardiac function deterioration, with CISAT-overexpressing hMPCs demonstrating enhanced efficacy, consistent with our previous findings. Hemodynamic analysis via pressure–volume (PV) loop measurements corroborated the echocardiographic data, with parameters such as ejection fraction, stroke work, cardiac output, dP/dt_min, and dP/dt_max indicating preserved cardiac function in the hMPCs+Ctrl group and optimal performance in the hMPCs+CISAT group compared to the vehicle group (Figure 6C–G). Collectively, these data provide robust evidence that transplantation of hMPCs, particularly those overexpressing CISAT, into MI hearts alleviate cardiac dysfunction and prevents left ventricular remodeling.

### 3.7. CISAT-Overexpressing hMPCs Promote Cardiac Vascular Regeneration and Suppress Endogenous Apoptosis

**Figure 7:**
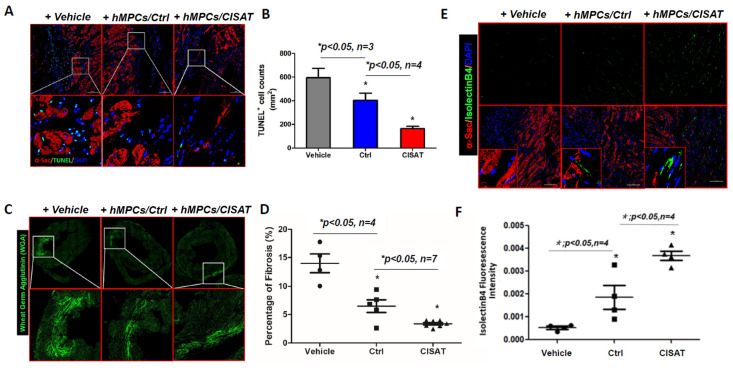
Transplantation of CISAT-overexpressing hMPCs in a murine MI model reduces apoptosis, attenuates cardiac fibrosis, and promotes endogenous vascular regeneration. (**A**) Representative immune-histochemistry images of TUNEL staining in the risk area of a murine MI model. TUNEL-positive cells (green) indicate apoptosis, α-sarcomeric actin (α-SA)-positive cells (red) mark cardiomyocytes, and DAPI (blue) stains nuclei. (**B**) Quantitative analysis of TUNEL-positive cells in the risk area. (**C**) Representative images of cardiac fibrosis assessed by WGA staining, with bright green areas indicating fibrotic regions in the heart. (**D**) Quantitative analysis of cardiac fibrosis, expressed as the percentage of fibrotic area relative to the whole heart. (**E**) Representative immuno-histochemistry images of IB4 staining for vascular structures in the risk area of the MI heart. IB4-positive cells (green) indicate vessels, α-SA-positive cells (red) mark cardiomyocytes, and DAPI (blue) stains nuclei. (**F**) Quantitative analysis of IB4 fluorescence intensity, with higher intensity reflecting increased vascular regeneration. The y-axis represents arbitrary units of IB4 fluorescence intensity. Each experimental group included at least 4 mice, with more than 20 images analyzed per slide. Data are presented as mean ± SEM. * *p* < 0.05, as calculated using one-way ANOVA followed by Tukey’s multiple comparison test.

Our findings demonstrate that transplantation of control hMPCs (hMPCs+Ctrl) and CISAT-overexpressing hMPCs (hMPCs+CISAT) into a mouse model of ischemia/reperfusion (I/R)-induced myocardial infarction (MI) improves cardiac structure and mitigates dysfunction. Given our in vitro evidence that CISAT protects hMPCs from apoptosis, we conducted terminal deoxynucleotidyl transferase dUTP nick-end labeling (TUNEL) staining to assess cardiac apoptosis in vivo. The results revealed a high density of apoptotic cells in the vehicle group (594.54 ± 78.89 cells/mm^2^ of cardiac area). In contrast, the hMPCs+CISAT group exhibited significantly fewer apoptotic cells (133.22 ± 44.28 cells/mm^2^), indicating a marked reduction in apoptosis in the risk area (Figure 7A,B). The hMPCs+Ctrl group also showed a significant decrease in apoptotic cell density (403.58 ± 70.88 cells/mm^2^) compared to the vehicle group (Figure 7A,B), consistent with our prior findings [18]. These results confirm that CISAT not only protects hMPCs from apoptosis in vitro but also inhibits endogenous cardiac apoptosis in vivo. Additionally, we evaluated cardiac remodeling using wheat germ agglutinin (WGA) staining, which allows clear visualization of cardiomyocyte borders and myocardial architecture and has been widely used to assess cardiac structural changes after injury. The hMPCs+CISAT group displayed significantly reduced fibrosis compared to controls (Figure 7C,D). To investigate the impact of CISAT-overexpressing hMPCs on cardiac vascular regeneration, we stained heart tissues with Isolectin B4 (IB4) conjugated to a green-fluorescent dye to visualize vasculature. Fluorescence imaging revealed significantly stronger capillary fluorescence intensity in the risk area of hMPCs+CISAT-treated hearts compared to the vehicle and hMPCs+Ctrl groups, indicating enhanced vascular regeneration (Figure 7E,F). This enhanced endogenous vascular regeneration may result from the microenvironment created by transplanted hMPCs+CISAT, which likely promotes neovascularization, supplying nutrients and oxygen to mitigate ischemic cardiac deterioration.

## 4. Discussion

In this study, we identified a novel lncRNA, termed CoPP-Induced and SFPQ-associated RNA transcript (CISAT), which is significantly upregulated in hMPCs following preconditioning with cobalt protoporphyrin (CoPP). Our findings demonstrate that CISAT, a specific transcript of the lnc-ANKMY1-5 gene, plays a critical role in mitigating cellular senescence and enhancing the therapeutic potential of hMPCs for myocardial repair. By interacting with the splicing factor SFPQ and modulating NRF2-related redox homeostasis and the p38 MAPK signaling pathway, CISAT promotes cell survival, proliferation, and migration while reducing senescence markers such as p16^INK4A^. These molecular effects translate to improved outcomes in an immune-deficient murine myocardial infarction model, with reduced fibrosis, enhanced angiogenesis, and preserved cardiac function following transplantation of CISAT-overexpressed hMPCs (as depicted in our graphic abstract). These results highlight CISAT as a promising therapeutic target to enhance the efficacy of hMPC-based regenerative therapies for heart disease.

The role of adult cardiac stem/progenitor cells (CPCs) in cardiomyocyte regeneration remains contentious, as studies using Cre-Lox lineage tracing in mouse models have shown that c-kit^+^ and sca-1^+^ CPCs contribute minimally to cardiomyogenesis [23,24,25,26], suggesting that their benefits in cardiac repair may limited to enhanced cell survival, secretion of protective cytokines, or promotion of vascular regeneration through mechanisms not fully elucidated. Challenges persist in leveraging CPCs for effective cardiac regeneration and cellular therapies for cardiovascular diseases. This study introduces novel evidence demonstrating that enhancing cell survival in vitro prior to transplantation significantly improves the therapeutic efficacy of engineered hMPCs in cardiac repair. Overexpression of lncRNA CISAT in hMPCs promotes cell survival, proliferation, migration, and rejuvenation of aging cells, collectively enhancing their therapeutic potential for cardiovascular treatment. Notably, transplantation of CISAT-engineered hMPCs protects against ischemia/reperfusion-induced cell death, enhances vascular regeneration, and improves cardiac function, evidenced by increased left ventricular ejection fraction, reduced left ventricular diastolic/systolic volumes, restored cardiac output, and attenuated adverse remodeling through decreased scar size and fibrosis. These findings underscore the potential of engineered hMPCs as an innovative and promising strategy for restoring cardiac function in ischemic heart disease and heart failure, reinforcing the value of adult human CPC-based therapies.

Redox-regulated signaling pathways are central to orchestrating cell survival and tissue remodeling in the injured myocardium [27,28,29]. Oxidative stress generated during ischemia–reperfusion activates a network of signaling cascades that critically determine progenitor cell viability and the trajectory of cardiac repair [30,31]. The transcription factor NRF2 serves as a principal regulator of endogenous antioxidant responses, coordinating the expression of cytoprotective genes that attenuate oxidative damage and preserve cellular homeostasis [32,33]. In this context, our observation that CISAT is upregulated in response to CoPP is consistent with established evidence that CoPP, a potent inducer of heme oxygenase-1 (HO-1), enhances the survival and functional integrity of cardiac progenitor cells (CPCs) under oxidative stress [10]. CoPP-mediated preconditioning has previously been shown to protect human cardiac stem cells (hCSCs) [10] and human embryonic stem cell-derived cardiomyocytes (hESC-CMs) [34] against ischemia–reperfusion injury through activation of the NRF2/HO-1 pathway, suppression of reactive oxygen species (ROS), and promotion of angiogenesis. The present study extends these findings by identifying CISAT as a downstream effector of CoPP signaling in human mesenchymal progenitor cells (hMPCs). The interaction between CISAT and the multifunctional RNA-binding protein SFPQ [35] suggests a novel regulatory mechanism whereby lncRNA-mediated modulation of gene expression counteracts oxidative stress and cellular senescence. Collectively, our findings propose that CISAT operates within a SFPQ/NRF2/p38 MAPK signaling axis to enhance the stress tolerance of CPCs, representing an additional regulatory layer through which noncoding RNA networks coordinate redox signaling during myocardial repair and regeneration.

As an alternative cell source, human-induced pluripotent stem cell-derived cardiomyocytes (hiPSC-CMs) have emerged as an established platform to study human cardiac signaling and stress responses [36,37]. Previous studies have demonstrated that redox-sensitive pathways, including NRF2-dependent antioxidant signaling [38,39] and MAPK/p38 pathways [40,41], are conserved in hiPSC-CMs and regulate cardiomyocyte maturation and injury responses. These findings suggest that the redox-regulated transcriptional mechanisms described in this study are likely conserved in human cardiomyocytes.

The observed decrease in CISAT expression in aging/senescent hMPCs, concomitant with elevated p16^INK4A^, underscores the link between lncRNA dysregulation and cellular aging. Senescence is a major barrier to the therapeutic efficacy of CPCs, as it impairs proliferation, migration, and paracrine signaling critical for myocardial repair [4]. Our gain-of-function studies demonstrate that CISAT overexpression reverses these senescence-associated phenotypes, reducing p16^INK4A^ expression and enhancing hMPC survival under oxidative stress induced by H_2_O_2_. This is consistent with the role of lncRNAs in regulating cellular redox homeostasis, as seen with lncRNAs like MALAT1 [15] and H19 [14], which modulate oxidative stress responses in other cell types [13]. The inhibition of p38 MAPK phosphorylation by CISAT further supports its anti-senescence function, as p38 MAPK activation is a hallmark of stress-induced senescence in progenitor cells [42]. By inactivating this pathway, CISAT likely mitigates apoptosis and promotes cell survival, providing a mechanistic basis for its cardioprotective effects.

Our previously published findings [18] demonstrated that transplanted hMPCs were successfully retained within the recipient myocardium, confirmed by human-specific HNA antibody staining. The majority of engrafted hMPCs adopted vascular cell fate (HNA^+^/vWF^+^ and HNA^+^/SMA^+^). Results from this study showed that the transplantation of CISAT-overexpressed hMPCs into a murine myocardial infarction model resulted in significant improvements in cardiac outcomes, including reduced fibrosis and enhanced angiogenesis, supporting the conclusion that hMPCs+CISAT transplantation facilitates myocardial repair and mitigates cardiac dysfunction. These findings align with prior reports that CoPP-pretreated CPCs improve graft survival and myocardial repair through paracrine mechanisms, such as VEGF secretion and recruitment of endogenous progenitor cells [10]. The enhanced angiogenesis observed in our study may be attributed to CISAT’s regulation of NRF2-mediated antioxidant and angiogenic gene expression, which supports endothelial cell survival and vessel formation. The preservation of cardiac function further suggests that CISAT-overexpressed hMPCs exert both direct and paracrine effects, potentially involving exosomal lncRNAs, as seen in other CPC studies [43,44]. However, the specific paracrine factors modulated by CISAT remain to be identified, and future studies should investigate whether CISAT is secreted in exosomes or influences exosome cargo in hMPCs.

Despite these promising findings, several limitations must be acknowledged. *First*, the study was conducted in an immune-deficient murine model, which may not fully recapitulate the immune responses and microenvironmental complexity of human myocardial infarction. *Second*, while CoPP effectively induces CISAT, its clinical applicability is limited by potential toxicity and non-specific effects, as noted with metalloporphyrins [45]. Alternative strategies, such as nanoparticle-based delivery systems or small-molecule activators of CISAT, could enhance translational potential. *Third*, we recognized limitation of hMSCs in scalability and cardiac lineage commitment compared to hiPSC-CMs and cardiac progenitors; the present study primarily focuses on the role of lncRNA in augmenting cell therapy efficacy for myocardial infarction. We propose that the identified lncRNA-mediated mechanisms may similarly apply to hiPSC-derived cardiac progenitors, warranting future investigation. *Fourth*, the precise molecular interactions between CISAT, SFPQ, and NRF2 require further characterization, including the identification of specific antioxidant genes targeted by this complex. *Finally*, the role of other versions of lnc-ANKMY1-5 transcripts in hMPCs remains unclear, and their potential compensatory or antagonistic effects should be explored.

Future research should focus on several key areas. *First*, elucidating the full spectrum of CISAT’s downstream targets using transcriptomic (e.g, RNA-Seq) and proteomic (e.g., Mass Spectrum) approaches will provide deeper insights into its regulatory network. *Second*, investigating CISAT’s role in other CPC populations, such as c-kit+ hCSCs, or hiPSC-CMs could broaden its therapeutic relevance. *Third*, developing targeted delivery methods for CISAT overexpression, such as viral vectors or CRISPR-based activation, may overcome the limitations of cell-based therapy. *Finally*, clinical studies are needed to validate CISAT’s therapeutic potential in human patients with heart disease, particularly in the context of autologous or allogeneic hMPC therapies following genetic modification.

## 5. Conclusions

In conclusion, our study identifies CISAT as a novel lncRNA that enhances the therapeutic efficacy of hMPCs by counteracting senescence and promoting survival, proliferation, and myocardial repair. By modulating NRF2-related redox homeostasis and p38 MAPK signaling through SFPQ interactions, CISAT represents a promising target for improving CPC-based regenerative therapies. These findings contribute to the growing understanding of lncRNAs in cardiac aging and repair, paving the way for innovative strategies to treat heart disease.

## Data Availability

The datasets generated during or analyzed during the current study are available from the corresponding author upon reasonable request.

## Supplementary Materials

The following supporting information can be downloaded at https://www.mdpi.com/article/10.3390/cells15060557/s1, Figure S1: Results from qPCR confirmed the overexpression (A) or down-regulation of CISAT in hMPCs with lentivirus expressing CISAT, shRNA against CISAT, or their vector control. Figure S2: Cell apoptosis assay for hMPCs expressing shRNA against CISAT or scrambled shRNA. Figure S3: Western blot analysis showing the upregulation of p16^INK4A^ in senescent hMPCs induced by doxorubicin (A), and down-regulation of p16^INK4A^ in hMPCs expressing shRNA against p16INK4A. Figure S4: Confocal imaging with RNA-FISH staining showed the majority of CISAT located in the nuclear region of cells. Table S1: List of primers used in this study. Table S2: List of antibodies used in this study.
