# Supplementary material for: CISAT, a CoPP-Induced lncRNA, Improves Cardiac Mesenchymal Progenitor Cell Survival and Myocardial Repair via SFPQ/NRF2/p38 Redox Regulation
Source: Cells. 2026 Mar 20;15(6):557. doi: 10.3390/cells15060557 (PMC13025675; doi:10.3390/cells15060557)
Supplement: Supplementary file 1 [file cells-15-00557-s001.zip › cells-4170470-supplementary.pdf]

Supplementary Figure S1: Results from qPCR confirmed the overexpression (A) or down-regulation of CISAT (B) in hMPCs with lentivirus expressing CISAT, shRNA against CISAT, or their vector control.

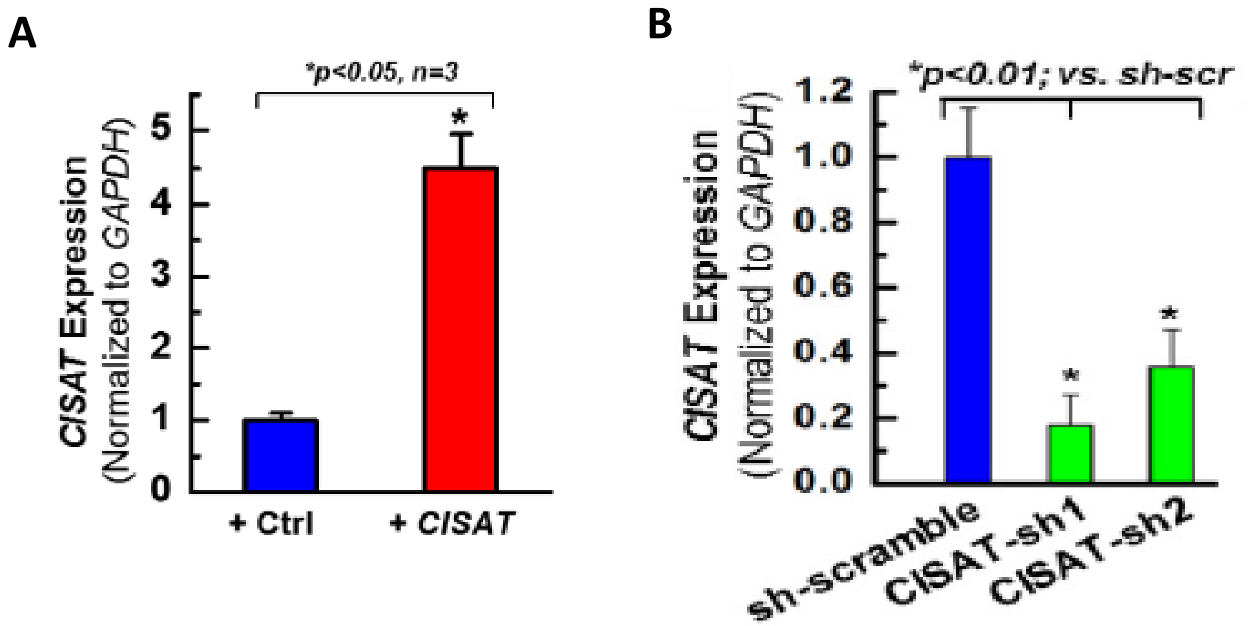

Supplementary Figure S2. **Cell apoptosis assay for knockdown CISAT in hCPCs by flow cytometry analysis with Annexin V-Fluor®647& propidium iodide (PI) double staining.**

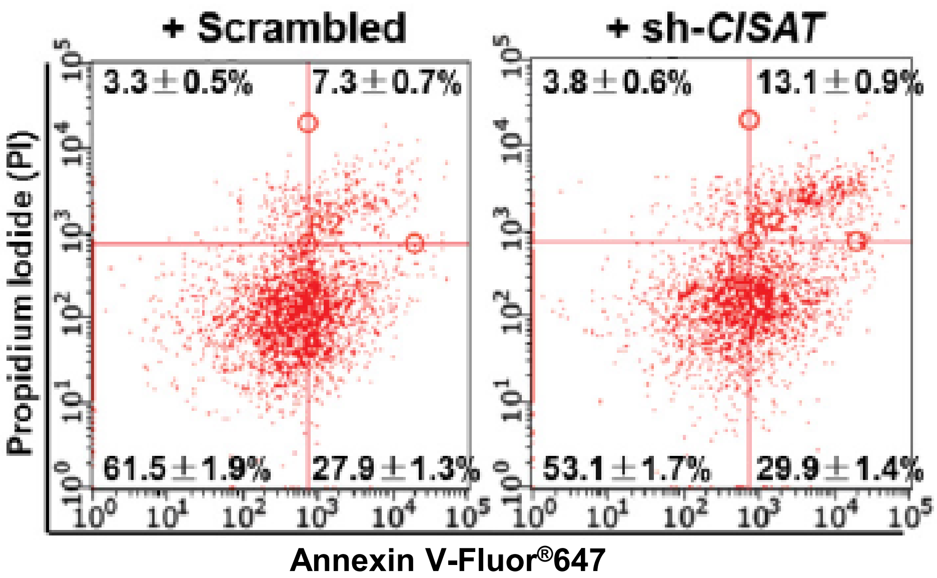

Supplementary Figure S3: Western blot analysis showing the upregulation of p16^{INK4A} in senescent hMPCs induced by doxorubicin (A), and down-regulation of p16^{INK4A} (B) in hMPCs expressing shRNA against p16INK4A.

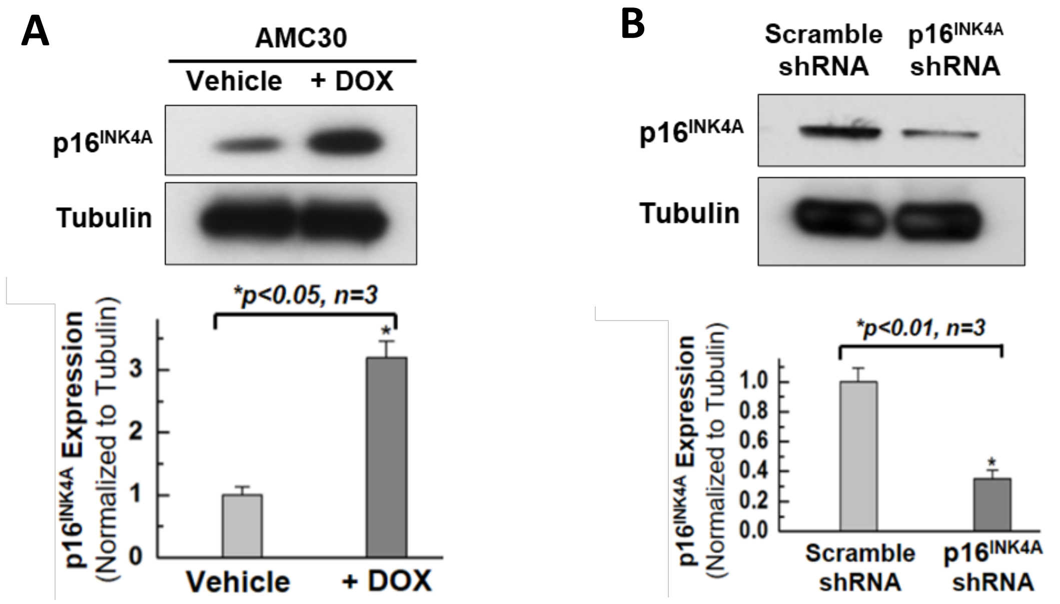

Supplementary Figure S4: Confocal imaging with RNA-FISH staining showed the majority of CISAT located in the nuclear region of cells.

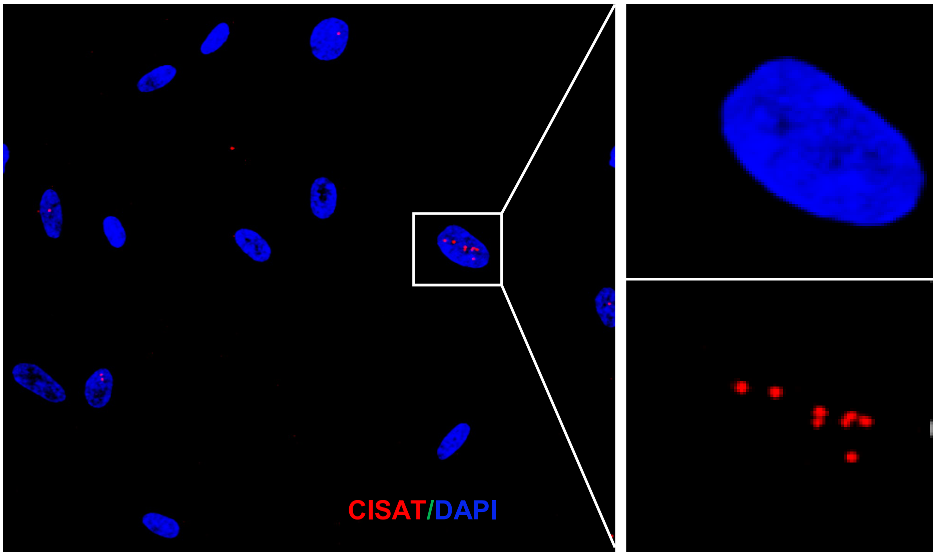

Supplemental Table S1. List of primers and their sequences used in this study.

Genes	Forward Primer	Reverse Primer
CDKN2A/p16	GCACATTCATGTGGGCATTT	GACTCAAGAGAAGCCAGTAACC
CDKN2C/p18	CGTCAATGCACAAAATGGATTTGG	GAATGACAGCGAAACCAGTTCGG
CDKN1A/p21	ATTAGCAGCGGAACAAGGAGTCAGACAT	CTGTGAAAGACACAGAACAGTACAGGGT
MAPK14/p38	GAAAGCAGGGACCTTCTCATAG	GTGCTCAGGACTCCATTTCTT
TP53/p53	GTACCACCATCCACTACAACTAC	CACAAACACGCACCTCAAAG
SFPQ	CGTGGAAGATCTACAGGGAAAG	CGAGGAGTTGTCGTCAGTAAG
NFE2L2 /NRF2	TGATTCTGACTCCGGCATTT	GCCAAGTAGTGTGTCTCCATAG
GPC1	CTATTGCCGAAATGTGCTCAAG	ACTCTCCACACCCGATGTA
OTOS	CACCTCTGACTTCTGGAACTATG	CACCATTCAGTCCTCCTGATAG
GAPDH	GGTGTGAACCATGAGAAGTATGA	GAGTCCTTCCACGATACCAAAG
BC104435	CCTTTCTGTGGACTGGGTTT	GGAGCTTTCCAGAGACCTTTAG
XLOC_004924	AGGAAGAAGGGAGAGAAGAAGA	CCTCCCAAAGTGCTAGGATTAC
RP11-393K12.2	CCTGGAAAGTGAACCACACA	GGAGCACCTAATAGGACACAAC
RP11-536K17.1	AGTGAGCTTTGAAGGGATCTTG	AGGAGTCTGGGTATGGGTTAG
RP11-71E19.5	TGACACCAATCCACCCATTT	GAGGCAGTGTGTTCTAGGTAAG
XLOC_004061	GGTCATCCACTCAGCTTCTT	TCACCATCATCCAGCCTTATTC
XLOC_002543/CISAT	CCGGTGAGAATATGTGGGTAAG	TGGAGCCCAGAAGTGATTTG
RP11-323H21.3	GTGCTGGGATTACAGGTATGAG	CTTTAAGGCAGGTGAGTAGGT
RP11-173L6.1	CTCACACTCGGATGCACTAAT	GGGCTGTAGGAAGCTCTTAAAC
XLOC_008334	GCTCCAGACTGCGTTCAAT	ATTGCTGGTCGGTCTCTTTATC
CTC-340A15.2	GCTGTGTGTCTCCAGAATCTT	ACCCAGTTTCTACTGCTTTCTT
LINC00473	GGCAGCCTCAGGTTACAAAT	AGGAGCAGGTAGGGAAATGA
RP11-476H24.1	CGCTCCAAACTCCAGCTTAT	CACAGGTTCTCCAAGGGTTAC
AC109586.1	AGACGATAGACCACCTCCTTAG	GAGTCCACTTTAGGTGCTTCTT
**Primers for different version of XLOC_002543 (CISAT) transcript**		
XLOC_002543 F1	CAGATTCCGCGAATCACAGT	
XLOC_002543 F2	GATGAGGTCTTGCTCTGTCATC	
XLOC_002543 F3	GGGAGCAGTAGCAAGGTTTAT	
XLOC_002543 F4	GGGAGCAGTAGCAAGGTTTAT	
XLOC_002543 F5	TCAAGTGGTGTGCCTGTTT	
XLOC_002543 F6	CACAAACGACAAGAGGTTCAAG	
XLOC_002543 F7	CCGGTGAGAATATGTGGGTAAG	
XLOC_002543 R1	TGGCTGAGAGGCAAGTTTC	
XLOC_002543 R2	TGACCCGATCCG	
XLOC_002543 R3	CAGCTAACTGTGACTACCTGAG	
XLOC_002543 R4	TATAAGGCTTGGCTGGGCTCACCT	
XLOC_002543 R5	CTCCATAGAACGGGCTTCTTAC	
XLOC_002543 R6	AGCCAACACACTTCCTGATAA	

Supplemental Table S2. List of primary antibodies used in this study.

Antigen	Catalog No.	IgG Type	Source
SFPQ	PLA0181	Rabbit	Sigma
$p16^{INK4A}$	92803	Rabbit	Cell signaling
BCL-2	2870	Rabbit	Cell signaling
Phospho-GSK3-β	9323	Rabbit	Cell signaling
Phospho-p65	3033	Rabbit	Cell signaling
SOD2	sc133254	Mouse	Santa Cruz
Catalase	SC50508	Rabbit	Santa Cruz
ERK1/2	SC135900	Mouse	Santa Cruz
Phospho-ERK1/2	9154	Rabbit	Cell signaling
MCL-1	5453	Rabbit	Cell signaling
p38	sc535	Rabbit	Santa Cruz
Phospho-p38	4511	Rabbit	Cell signaling
NRF-2	ab62352	Rabbit	Abcam
Phospho-NRF-2	ab76026	Rabbit	Abcam
PRDX3	10664-1-AP	Rabbit	Proteintech
Isolectin IB4 Conjugates	I21411	N/A	Thermo Fisher
α-Sarcomeric actin	A2172	Mouse	Sigma
Tubulin	2144	Rabbit	Cell signaling
GAPDH	2118	Rabbit	Cell signaling
